# Paper-Based Biosensors: Frontiers in Point-of-Care Detection of COVID-19 Disease

**DOI:** 10.3390/bios11040110

**Published:** 2021-04-07

**Authors:** Riccarda Antiochia

**Affiliations:** Department of Chemistry and Drug Technologies, Sapienza University of Rome, P.le Aldo Moro 5, 00185 Rome, Italy; riccarda.antiochia@uniroma1.it

**Keywords:** paper-based-biosensor, COVID-19, POC diagnostics, lateral flow immunoassay, nucleic acid later flow assay, paper-based microfluidics

## Abstract

This review summarizes the state of the art of paper-based biosensors (PBBs) for coronavirus disease 2019 (COVID-19) detection. Three categories of PBB are currently being been used for severe acute respiratory syndrome coronavirus 2 (SARS-CoV-2) diagnostics, namely for viral gene, viral antigen and antibody detection. The characteristics, the analytical performance, the advantages and drawbacks of each type of biosensor are highlighted and compared with traditional methods. It is hoped that this review will be useful for scientists for the development of novel PBB platforms with enhanced performance for helping to contain the COVID-19 outbreak, by allowing early diagnosis at the point of care (POC).

## 1. Introduction

Coronavirus disease 2019 (COVID-19) is the third coronavirus epidemic of the 21st century caused by SARS-CoV-2 (severe acute respiratory syndrome coronavirus 2), a highly contagious human coronavirus, which emerged in December 2019 in Wuhan, China, and rapidly spread worldwide, with almost 2,750,000 deaths reported [1,2,3,4,5].

The transmission of the virus occurs by respiratory droplets and aerosols between people. Current studies confirm that both symptomatic and asymptomatic infected people can be contagious [6,7]. Therefore, the World Health Organization (WHO) guidelines suggest physical distancing, wearing a mask, avoiding crowds and keeping rooms well ventilated [8,9,10].

Hence, the development of fast, sensitive and low-cost diagnostic devices to track the spread of the virus by accurate screening of people is a priority need, so that proper isolation of infected people and treatment of patients can be facilitated [11,12,13,14,15].

**Figure 1:**
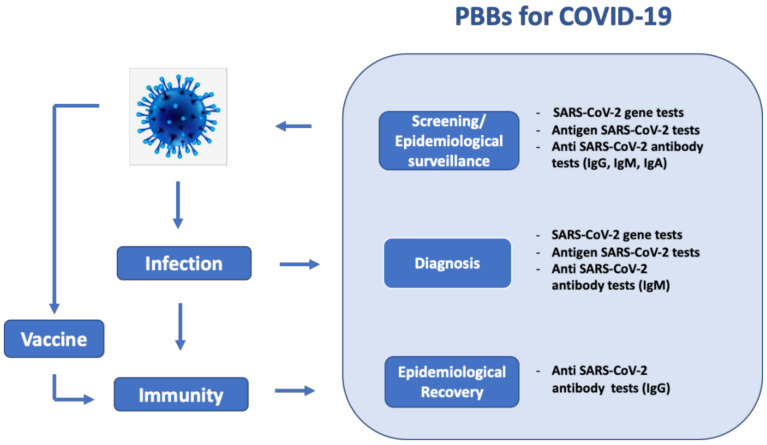
The important role of paper-based biosensor (PBB) diagnostics tests in the context of the Coronavirus disease 2019 (COVID-19) pandemic for screening, diagnosis and epidemiological recovery/surveillance.

The diagnostic tools available so far are based on (a) detection of the RNA virus (viral gene detection), (b) detection of a part of the virus (antigen detection test) and (c) detection of human antibodies (serological test), as schematized in Figure 1. Among these methods, viral gene detection by reverse transcription polymerase chain reaction (RT-PCR) has been found to be the most reliable technique [16,17,18]. It is a well-established commercial technique with high sensitivity, thanks to signal amplification, and large scalability to thousands of detection kits. It has the limitation of being time-consuming (2–5 h), expensive and limited to specialized laboratories, and, therefore, is not suitable for massive population testing [19]. The other molecular biology technique, namely reverse transcription loop-mediated isothermal amplification (RT-LAMP), is a more recent technique, where the amplification is conducted at a single temperature and does not need specialized laboratory facilities but still requires trained staff to operate the processing steps [20,21,22,23]. Therefore, both methods are not usable at the point of care (POC) [24].

The antigen detection test is based on the detection of viral antigens by using specific antibodies. It is rapid (5–15 min), low-cost, usable at the POC and, therefore, is ideal for massive COVID-19 detection. Unfortunately, to date, few studies have been published and only a limited number of tests are available on the market.

Serological tests allow the detection of the antibodies produced by the infected person during the disease. The rapid and low-cost biochemical enzyme-linked immunosorbent assay (ELISA) is the most commonly used method to detect the specific antibodies in a patient’s blood [25,26]. Unfortunately, this method requires the use of specialized laboratories and well-trained personnel, and it is not usable at the POC. Several serological kits are already on the market to cope with the emergency detection of COVID-19 antibodies. However, they still show limited sensitivity, with the possibility of a large number of false positive results because of the potential cross-reactivity [27,28].

Of course, the three diagnostic methods give different information: viral gene and antigen detection tests tell if a person is currently infected, whereas serological tests can determine prior infections. Although, in a recent study, the ELISA test could detect antibodies as early as 3 days after the development of the first symptoms [25], serological tests are generally used for rapid screening of post-symptomatic patients who have already developed the immune response [26,27,28,29] for epidemiological surveillance and post-vaccine immunization studies (Figure 1).

It is clear that a rapid, low-cost, portable test that can detect the virus with high sensitivity and specificity would be a great advance. An ideal diagnostic tool should fulfil the so-called ASSURED (affordable, sensitive, specific, user-friendly, rapid and robust, equipment-free and deliverable to end users) criteria guidelines provided by the World Health Organization (WHO) for POC testing [30,31]. Tools that satisfy the ASSURED criteria primarily aim to provide same-day diagnosis and facilitate immediate decision-making.

Among the POC methods actually used for COVID-19 detection, paper-based biosensors (PBBs) [32,33,34] and affinity-based biosensors (ABBs) [35,36] represent challenging alternatives, as they can be used by the patients themselves, coupling a fast response time with a good level of sensitivity.

In particular, PBBs are revolutionizing POC approaches for health applications by providing low-cost and disposable tools which can be utilized in remote settings. Colorimetric, electrochemical, fluorescence and chemiluminescence methods are typically used as detection techniques, relying on either external equipment for analysis or visual colorimetric read-outs, with the latter being prone to errors and usually not providing quantitative results [37].

The main focus of this review is to provide an overview of current PBBs for COVID-19 detection. The typical configuration, the principles and a general classification are presented first. Successively, the PBBs recently published in the literature are critically reviewed and divided into three categories, depending on the target analyte: (a) viral genes, (b) viral antigens or (c) antibodies. A critical comparison of their analytical performances in terms of detection limits, sensitivity, specificity and time of analysis is reported. Finally, future perspectives and key challenges of PBB development and applications will be discussed.

## 2. Paper-Based Biosensors

PBBs have been widely investigated for developing POC devices, thanks to their simplicity, affordability and ease of use [32,33,34]. The use of nanoparticles (NPs) as labels plays a crucial role in the design and development of a PPB strip. The performance of these devices is directly affected by the choice of NP and the corresponding detection method [38,39,40].

**Figure 2:**
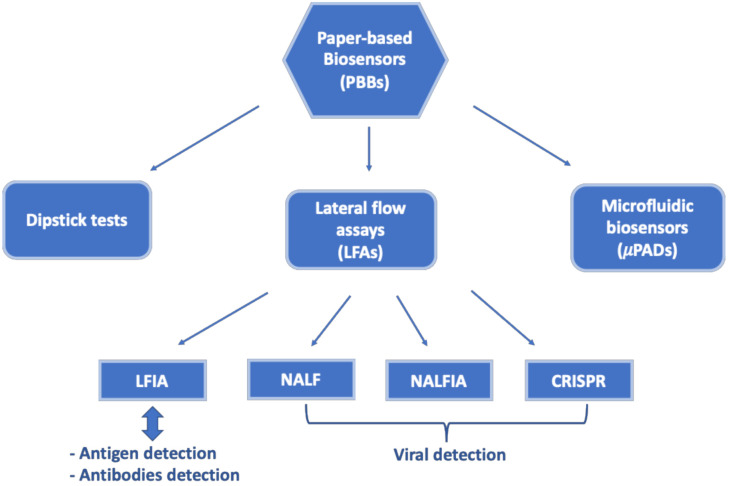
Schematic classification of PBBs. Abbreviations: LFIA = lateral flow immunoassay; NALF = nucleic acid lateral flow; NALFIA = nucleic acid lateral flow immunoassay; CRISPR = clustered regularly interspaced short palindromic.

They can be classified into three categories: dipstick tests, lateral flow assays (LFAs) and microfluidic biosensors (μPADs), as schematized in Figure 2.

### 2.1. Dipstick Tests

**Figure 3:**
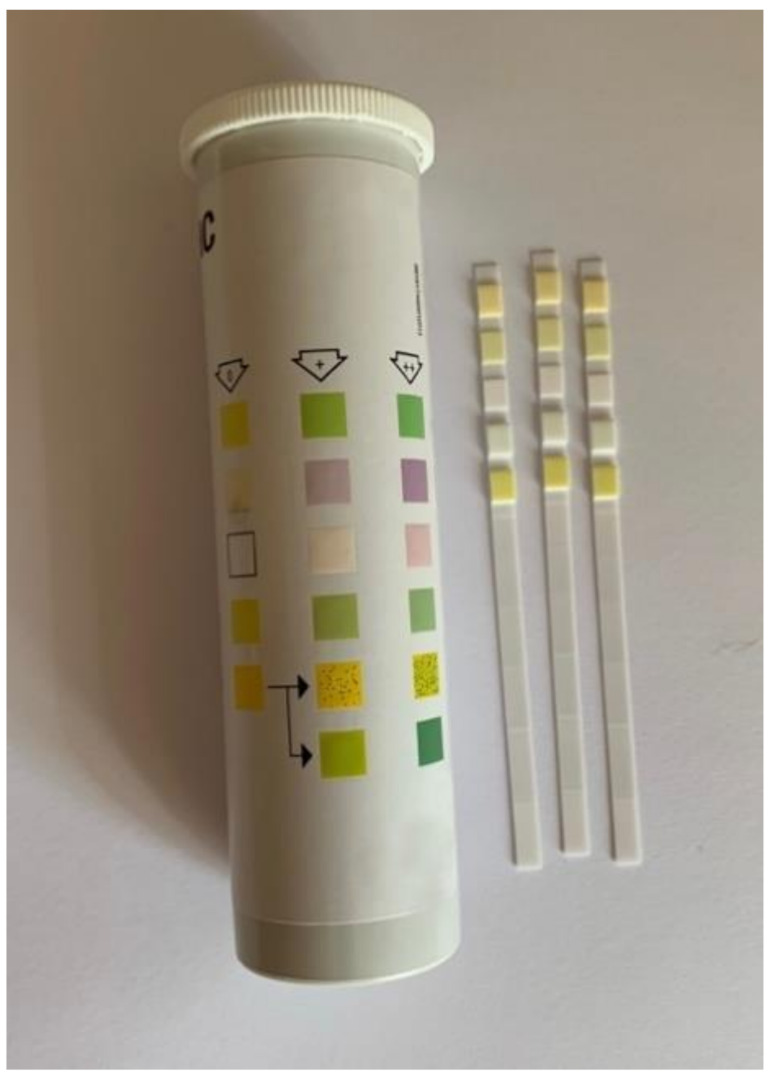
Commercial urine dipstick. It contains up to 10 different chemical pads which change color when it is immersed in and then removed from a urine sample, allowing semi-quantitative analysis of analytes such as glucose, ketones, bilirubin and pH.

A dipstick test is a chemically sensitive strip of paper originally used to identify one or more constituents of urine by immersion (Figure 3). Successively, it has been used as simple diagnostic tool consisting of immobilized reagents for qualitative analysis for the detection of several bioanalytes, such as glucose, uric acid, proteins, biomarkers, pathogens, etc. The main disadvantages are inaccuracy and its compatibility with optical detection only [41].

### 2.2. Lateral Flow Assays

A lateral flow assay (LFA) is a particular self-operating biosensor that performs rapid assays on a membrane in a chromatographic manner. The recognition layer is realized onto the surface of a porous membrane, usually a nitrocellulose membrane, suitable for maintaining the flow of the sample and reagents by capillarity and holding specific recognition elements, confined in particular zones of the membrane itself [42,43,44,45,46]. The introduction of NP labels has made LFAs more sensitive and selective. It is well known that NPs have unique electrical, optical, magnetic and thermal properties, which enable them to produce a specific signal when combined with different analytes. Colloidal gold, fluorescent NPs, surface enhanced Raman scattering (SERS) nanomaterials, magnetic NPs and carbon nanomaterials are commonly used in LFA applications to produce different kinds of signal for quantitative or semi-quantitative analysis [47].

Lateral flow biosensors (LFBs) are a leading technology in POC diagnostics, thanks to their simplicity, rapidity and low cost. Moreover, the LFA architecture is adaptable to multiplex testing. Therefore, it represents, in principle, an ideal solution to the continuous need for POC multiplexing analysis. Unlike other multiplex testing methods, such as PCR and ELISA, which require expensive instrumentation, a long analysis time and skilled personnel, multiplexed LFBs are promising POC candidates that perfectly fulfill the ASSURED criteria [48,49,50,51].

Depending on the recognition elements used, a LFA can be divided into three categories (Figure 2): lateral flow immunoassays (LFIAs), nucleic acid lateral flow assays (NALFs) and nucleic acid lateral flow immunoassays (NALFIAs), depending on whether antibodies/antigens, nucleic acid or both are used as recognition elements for the detection of antigens/antibodies or amplicons, respectively; the latter are formed during the amplification process by PCR or loop-mediated isothermal amplification (LAMP) [43].

#### 2.2.1. Lateral Flow Immunoassays

LFIAs, also called immunochromatographic tests, are well known for their use as an in-situ platform for pregnancy tests for human chorionic gonadotropin (HCG) protein detection [52]. However, they have a wider scope of application in medicine for the diagnosis and prognosis of several diseases, such as infections, tumors, cardiology or neurological-related disorders and allergies [53].

The main drawback of the conventional LFIA is its poor sensitivity [54,55,56,57]. Several methods to improve LFA sensitivity have been used, such as enrichment or concentration methods, divided into on-strip and off-strip pre-enrichment, temperature–humidity control, enzyme-based signal enhancement, etc. [58,59,60,61]. However, research on simplified and low-cost methods is still needed.

**Figure 4:**
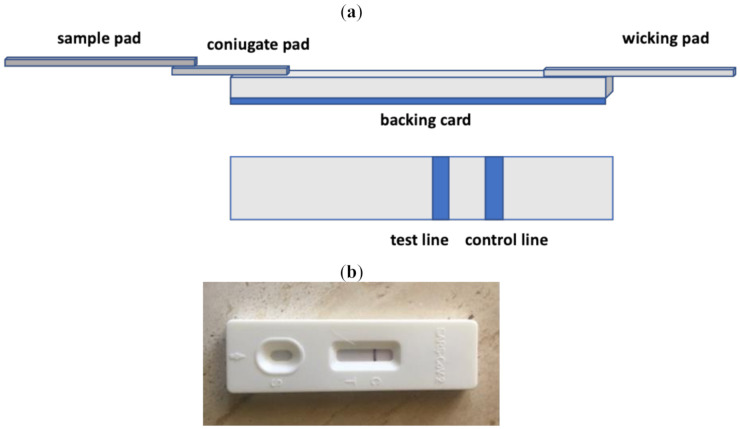
Schematic representation of a lateral flow immunoassay (LFIA) strip (**a**); a commercial lateral flow immunoassay test (**b**). When a sample containing the analyte is applied to the sample pad, a specific reagent with the target analyte migrates to the conjugate pad, forming a colored line in both the test and control lines. If no analyte is present in the sample, only the control line is visible.

The main parts and the mechanism of a LFIA strip are shown in Figure 4, together with a commercial LFIA strip. The sample solution is added to a “sample pad” at one end of the strip and flows by capillarity through a polymeric membrane strip, on which biomolecules, which are able to interact with the analyte, are immobilized. Finally, it reaches the “conjugate pad”, which contains the specific antibodies to the target analyte, usually conjugated with colored or fluorescent NPs [62,63,64,65,66,67,68,69]. The target analyte/conjugated antibody complex then flows along the strip through the nitrocellulose membrane, where specific biological components (antibodies or antigens) are fixed in particular zones, called “test lines”. A control reagent is immobilized in a second line, called the “control line”, further along the membrane. The analyte is either captured at the test line, producing a color change, visible to the naked eye or by using a specific reader, or continues to migrate until reaching the absorbent “wicking pad” at the other end of the strip. A proper response on the “control line” indicates the assay has run successfully. Visual inspection of the colors at the test and control lines allows qualitative or semi-quantitative analysis. For quantification, optical strip readers, such as cameras processed by specific imaging software, are necessary for measurement of the color intensities [70,71,72]. The “wicking pad” at the end of the strip allows one to maintain the flow rate of the liquid and to stop backflow of the sample solution. An array format consisting of additional test lines of specific antibodies to different analytes can be used to test multiple analytes simultaneously under the same conditions [73]. All these components are fixed over a “backing card”, used as a support platform.

Two basic formats of LFIA, namely the sandwich and competitive formats, have been developed.

**Figure 5:**
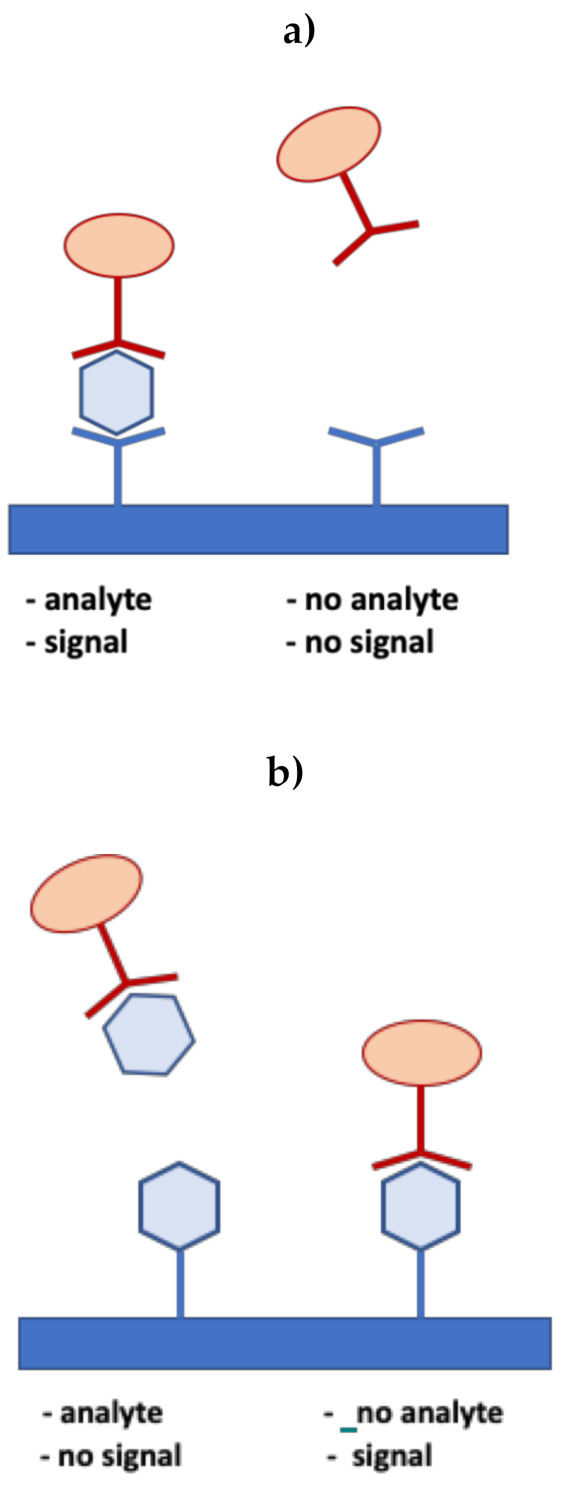
Sandwich (**a**) and competitive (**b**) assay formats. In a sandwich assay, a positive signal indicates the presence of the analyte, while in a competitive format, the signal on the test line means no analyte is present.

The sandwich test is used for analytes with more than two antigenic sites, which can be bound to two different kinds of antibody simultaneously, such as the human chorionic gonadotropin (hCG) used in pregnancy tests [52]. In the sandwich format, the analyte is captured between two complementary antibodies, as schematized in Figure 5a. One of these antibodies (the reaction antibody) is conjugated with labels and is held at the conjugate release pad, while the other antibody (the detection antibody), whose function is to capture the target antigen, is immobilized at the test line on the membrane. In this format, the function of the control line is to capture any excess labeled antibody. The signal intensity at the test line is directly proportional to the amount of analyte present in the sample.

For the detection of smaller analytes with a single antigenic determinant, which cannot bind two antibodies simultaneously, competitive tests, based on one single type of antibody, are utilized [74]. The analyte molecule and the antibody–nanoparticle conjugate are typically linked to the test line and the conjugate pad, respectively. When the target analyte is present in the sample, it binds to the conjugate, thus preventing it from binding to the analyte at the test line. Otherwise, in the absence of the analyte, the conjugates bind to the analyte linked to the test line, producing a signal, as shown in Figure 5 b. Therefore, in the competitive format, the signal intensity is inversely proportional to the amount of analyte present in the sample. As in the sandwich format, the control line binds the conjugate with or without the analyte, indicating that the assay is working correctly. In summary, the presence or absence of a visible signal at the test line indicates a positive result in the sandwich and competitive format, respectively.

#### 2.2.2. Nucleic Acid Lateral Flow Assays

The accurate definition of a NALF does not include any immunoreagent on the nitrocellulose membrane. The recognition element is represented by a DNA or RNA probe. The signal originates from a sandwich hybridization reaction occurring at the test line, to which a DNA probe is usually bound by a terminal biotin, which interacts with an avidin molecule on the nitrocellulose [75,76,77,78]. As in LFIAs, signaling moieties are usually oligonucleotide-decorated gold NPs, or simply fluorescent dyes that modify the tag sequence [79,80,81,82].

A signal amplification is required for the detection of nucleic acids. Whilst PCR is the most commonly reported method of amplification used in combination with lateral flow assays [83,84], an increasing number of papers are starting to report the combined use of isothermal amplification with lateral flow detection, thus approaching the standard required by the ASSURED devices, usable at the POC [85,86].

#### 2.2.3. Nucleic Acid Lateral Flow Immunoassays 

**Figure 6:**
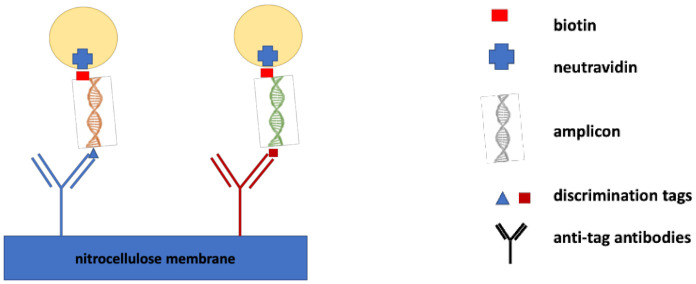
Schematic representation of a nucleic acid lateral flow immunoassay (NALFIA) test: neutravidin molecules adsorbed onto gold nanoparticles (AuNPs) detect biotin-labeled amplicons. The discrimination tag is recognized by its specific antibody, immobilized onto a nitrocellulose membrane.

A NALFIA is a hybrid format incorporating the selectivity of Watson–Crick base pairing, the sensitivity of molecular techniques and a known immunochromatographic assay. It is designed to test the presence of an amplified nucleic acid sequence specific to the target analyte, using primers with two different tags (sandwich format). Recognition of the analyte is done by binding to a tag-specific antibody, previously sprayed on the nitrocellulose membrane, and another tag-specific antibody conjugated to colored nanoparticles enabling visualization, as schematized in Figure 6 [87,88].

Both NALF and NALFIA methods combine the power of enzymatic exponential amplification of the target gene sequence with the sensitivity and ease of use offered by the LFIA technique [76].

### 2.3. Paper-Based Microfluidics 

Microfluidics is a multidisciplinary technology dealing with the flow of liquids inside micrometer-sized channels. It can be used to improve existing diagnostic tools, making them more accurate and efficient and less costly.

**Figure 7:**
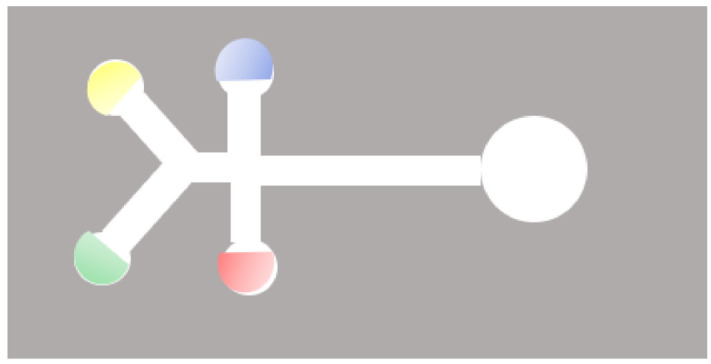
Schematic of a two-dimensional paper-based microfluidic device. Cellulose-based filter paper is precisely cut to produce hydrophilic microchannels which form five detection zones, each for the colorimetric detection of a specific analyte.

Paper-based microfluidics, also referred to as microfluidic paper-based analytical devices (μPADs), are diagnostic systems made of patterned paper, on which a small volume of fluid will move by capillarity, through multi-channel designs within the paper substrate [89,90,91,92]. An example is shown in Figure 7.

Paper-based microfluidics are particularly suited for multiplex testing for various biological samples including whole blood, serum, urine and nasal swabs [93,94]. The μPADs are compatible with different detection modes, such as colorimetric, electrochemical, chemiluminescence and electrochemiluminescence detection, and with automated sample processing towards the envisaged ASSURED device. 

Furthermore, paper is suitable for clinical analysis because it is biologically compatible and able to easily absorb reagents. Moreover, its normally white background provides a contrast for color-based detection methods. Paper is also light, produced at low cost and available worldwide. With these properties, it represents an ideal material to build eco-friendly portable devices.

Several 3D paper-based microfluidic biosensors have been realized for the detection of proteins or nucleic acids at the POC [91,95,96], based on fluorescence or colorimetric detection approaches. Integrated devices, obtained by combining μPADs with camera phones or other imaging devices that are able to quantify the colorimetric results and to transmit the measured data to trained physicians, have been realized and represent the first applications of telemedicine for inexpensive monitoring of human health, which is particularly important in developing countries [97].

## 3. Applications of Paper-Based Biosensors for COVID-19 Detection

### 3.1. SARS-CoV-2 Gene Detection

Even though RT-PCR remains the gold standard method for diagnosis of suspected COVID-19 cases, LFAs are becoming a more practical alternative, as they allow simultaneous detection of multiple regions of nucleic acids in an easy-to-use, rapid and inexpensive test.

The first NALF assay for the simultaneous detection of three regions of the SARS-CoV-2 genome (RdRp, ORF3a and the nucleocapsid (N)-protein gene) after RT-PCR amplification has been developed by Yu and co-workers [98]. It allows the detection of SARS-CoV-2 in 30 min with a limit of detection (LOD) of 10 copies/test for each gene. A fluorescence signal is obtained after the hybridization of each specific probe with the respective PCR products. The simultaneous detection of the three genes allows one to avoid cross-reactivity with other coronaviruses and possible false negative results caused by possible mutations in the SARS-CoV-2 genome. The assay was tested in 162 clinical samples of both nasopharyngeal swabs and sputum from COVID-19 patients, and showed very high concordance with the commercial assay. A PCR machine is necessary for the amplification of the genomic copies, thus limiting the possibility of the NALF assay to be developed into a POC test.

In another work developed by Zhu et al. [99], the amplification step was realized by using a multiple reverse transcription LAMP, coupled with a NP-based lateral flow test strip, for COVID-19 diagnosis. Briefly, two target sequences (ORF1ab and the N gene) were amplified utilizing two LAMP primer sets in an isothermal reaction. Fluorescein (FITC)-, digoxin- and biotin-attached duplex amplicons were produced by RT-LAMP in presence of FITC-, digoxin- and biotin-labeled primers on the nitrocellulose membrane. Successively, the ORF1ab and N genes of SARS-CoV-2 were simultaneously detected by NALFIA through immunoreactions and biotin/streptavidin interaction. The LOD was 12 copies per reaction with no cross-reactivity. The assay was validated using clinical oropharynx swab samples from 33 patients infected with SARS-CoV-2 and 96 non-SARS-CoV-2 infected patients, showing high concordance with a commercial RT-PCR method.

In order to meet the ASSURED criteria, several studies have been conducted, focusing on signal amplification by functionalization of the DNA probes, for example, with fluorescence NPs, rather than target amplification by PCR and LAMP [100].

In this context, Wang et al. [101] presented an amplification-free, rapid and easy method for SARS-CoV-2 nucleic acid detection, usable at the POC. It is basically a nucleic acid immunoassay, implemented on a lateral flow strip (NALFIA) for fluorescence detection of viral RNA in less than 1 hour. The assay is based on capture of the RNA–DNA hybrids. DNA probes are used to bind three regions of the SARS-CoV-2 genome (ORF1ab, envelope protein and the N-protein gene), while a monoclonal antibody, previously labeled with fluorescent NPs, is added to bind to the double-stranded DNA–RNA hybrids. Therefore, only an immunofluorescence lateral flow strip and a fluorescence device are required for the test. The contamination by amplicons is avoided thanks to the absence of nucleic acid amplifications, unlike other LFA methods for nucleic acid detection. The assay showed a very good sensitivity (LOD of 500 copies per mL for clinical throat swab samples), the absence of cross-reactivity and high robustness, thanks to the high affinity of the monoclonal antibody to DNA–RNA hybrids. Moreover, the proposed assay can meet the demand for POC technologies and can be adapted for the detection of other RNA viruses.

Clustered regularly interspaced short palindromic repeats (CRISPR), a sophisticated biotechnological technique used for in vitro detection of nucleic acids, has been recently combined with LFA principles in one assay and utilized as an in situ diagnostic tool for rapid detection of the SARS-CoV-2 virus [102,103,104,105]. This method is based on some nuclease enzymes, which are programmed to cut certain viral RNA sequences and are successively amplified by an isothermal amplification step, thus avoiding the use of expensive thermocycles. Most studies on CRISPR-based SARS-CoV-2 detection use Cas12 or Cas13 nuclease enzymes for specific recognition of the viral sequence, which results in the cleavage of fluorescent or lateral flow reporter ssDNA.

As for Cas12-based technologies, Broughton et al. [106] adopted the DNA Endonuclease-Targeted CRISPR Trans Reporter (DETECTR), a Cas12a-based rapid lateral flow-based platform for SARS-CoV-2 detection. This method takes place 30 or 45 min to complete without and with an RNA extraction step, respectively, and requires an RT-LAMP amplification step in the N and E genes of SARS-CoV-2. The ssDNA cleavage activity of Cas12a allowed the detection of the amplicons, using fluorescence and lateral flow readout, with a limit of detection (LOD) of 10 copies/µL. The test was validated in 82 clinical samples extracted from nasopharyngeal swabs, showing 95% sensitivity and 100% specificity, compared with the RT-PCR assay recommended by the US Centers for Disease Control and Prevention (CDC). The assay requires very simple components and takes 45 min to occur. However, the monitoring of fluorescence signal requires a specialized device, while a visual inspection would eliminate this issue. 

To this end, Wang et al. [107] realized a sensitive and rapid CRISPR/Cas12a-based -detection method with a naked eye readout, named *CRISPR/Cas12a-NER*. A ssDNA reported labelled with a quenched green fluorescent molecule was added and then cleaved by Cas12a, in presence of nucleic acid of SARS-CoV-2. The fluorescence signal is detectable with the naked eye under blue light at 485 nm. An amplification step based on reverse transcription-recombinase aided amplification (RT-RAA) was used to obtain enough DNA for Cas12a-mediated detection. This assay was tested on 31 clinical samples, showing a 100% sensitivity and 100% specificity, compared to the WHO-recommended RT-PCR assay.

Another portable and sensitive CRISPR-Cas12 based device for COVID-19 was developed by Lucia and co-workers. [108]. Briefly, the SARS-CoV-2 fragments corresponding to the RdRp, ORF1b and ORF1ab genes were synthesized as complementary DNA and treated with PCR to generate DNA templates, successively transcribed into RNA. An isothermal amplification step (Recombinase Polymerase Amplification, RPA) was carried out in one step, for 30 min at 42 °C, followed by generation of the CRISPR-detection complex reaction for 10 min at room temperature. Both fluorescence and lateral flow readers were utilized, by using ssDNA reporters labeled with carboxyfluorescein and biotin, respectively. The sensitivity of the two assays proved to be similar (a LOD of about 10 copies/μL), demonstrating that the detection system can be made portable without a significant loss in sensitivity. The assay was tested on saliva samples from a healthy donor spiked with synthetic SARS-CoV-2 RNA fragments, showing promising results.

As for Cas13-based technologies, recently, Zhang et al. [109] reported the SHERLOCK technology for rapid detection of SARS-CoV-2, a specific high-sensitivity enzymatic reporter unlocking method based on a CRISPR/Cas13 nucleic acid detection technique. Again, an isothermal amplification method (Recombinase Polymerase Amplification, RPA) is required before starting the reaction in order to improve the sensitivity of the method. The production of quantifiable signals is obtained thanks to the presence of the ssRNA coronavirus genome in Cas13-activated samples. The assay can be read out in less than 1 h using a dipstick and showed a LOD of 10–100 viral RNA copies/µL.

The first Food and Drug Administration (FDA) - authorized application of CRISPR-Cas systems is another SHERLOCK assay, developed by combining RT-LAMP and Cas13a-based detection steps [110]. The LOD resulted to be lower in this case, 6.75 copies/µL, but the test showed high sensitivity and high specificity for SARS-CoV-2. The kit requires only standard laboratory equipment and a fluorescence reader. It is commercially available and represents the first and unique example of application of a CRISPR system for infectious disease diagnostics.

The use of isothermal amplification methods and lateral flow readout in CRISPR-based diagnostic studies meets the need not to use complicated equipment. However, PCR technique is more sensitive and can be used with widely available reagents, and the fluorescence readout is more reliable than the lateral flow readout. Therefore, attempts are in progress to use small, portable, battery-powered and inexpensive thermocyclers and fluorescence visualizers [111].

A new technology called Prophylactic Antiviral CRISPR in huMAN cells (PAC-MAN) was designed not only to identify the presence of the virus but also for viral inhibition. In this case, the Cas13 protein identifies and cuts a specific sequence of the viral RNA encoding for an essential protein for virus replication. By cutting the segment, the life cycle of the virus is blocked, thus stopping the infection. This technology, developed by scientists of Stanford University for the SARS-CoV-2 virus and published as a preprint [112], was already being studied against influenza viruses when the COVID-19 emergency broke out. The authors screened a group of CRISPR RNAs (crRNAs) targeting conserved viral regions and identified sequences for cleaving SARS-CoV-2. A set of six crRNAs targets more than 90% of all coronaviruses. The authors demonstrated that the PAC-MAN approach can effectively degrade SARS-CoV-2 and influenza A virus (IAV) sequences in human epithelial lung cells. The cells were analyzed through flow cytometry and microscopy. Unfortunately, this potentially therapeutic method is still far from applicability, as tests on animal and human models are missing to date. Furthermore, a vector capable of transporting Cas13 within cells has yet to be identified. For therapeutic use, the CRISPR components must be delivered into the cells by using effective delivery systems, such as liposomes, polymers or lipid nanoparticles. At the moment, the PAC-MAN strategy represents a potentially powerful new approach to inhibit pan-coronavirus functions and replication, but it not usable at the POC. Other studies are necessary to couple this method with lateral flow assay strips for virus identification.

The main analytical characteristics of the reported PBBs for SARS-CoV-2 gene detection, also including CRISPR methods, are summarized in Table 1.

### 3.2. SARS-CoV-2 Antigen Detection

Although a large number of LFIAs for SARS-CoV-2 detection are in development, the majority of them are serological tests for antibody detection, with only a small number for antigen detection. Unlike serological tests, which reveal previous exposure to SARS-CoV-2, the antigen assays detect actual viral infection, as PCR-based methods. Although antigen tests in ELISA format show better sensitivity and higher throughput, thanks to the possibility of simultaneous measurements, antigen tests operating on LFA strips are rapid, inexpensive, easily usable at the POC and offer a scalable solution, especially in low-resource countries.

Diao et al. [113] proposed a LFIA for detecting the nucleocapsid protein of SARS-CoV-2 in nasopharyngeal swabs and urine samples within 10 min. The assay utilizes mouse anti-N antibodies and goat anti-rabbit IgG antibodies to create the test and control lines, respectively. As signal particles, anti-N rabbit IgG labeled with carboxylate-modified polystyrene Europium (III) chelate microparticles are used. The fluorescent results are read by an immunofluorescence analyzer.

The assay was compared with nucleic acid testing, showing a sensitivity of 68% and a specificity of 100%.

In another work, a dipstick test has been realized by Grant and co-workers for SARS-CoV-2 nucleocapsid N antigen detection [114]. The dipstick test consists of a nitrocellulose membrane and a wick pad, without sample and conjugate pads (Figure 3). Commercially available polyclonal antibodies are utilized in this study, so cross-reactivity with other coronaviruses is possible. Latex particles were conjugated to the respective antibodies and the concentration of the conjugates was determined by measuring the absorbance at 560 nm for red and 660 nm for blue. The LFIA was tested in a buffer and showed a LOD of 0.65 ng/mL using both an optical reader and visual reads. The present work cannot be used in a commercially available product, but further studies will allow the use of this assay format with more specific antibody pairs in commercial assays.

Although there is an urgent need for LFIA SARS-CoV-2 antigen tests, a limited number of tests are available on the market. The first test, the Panbio COVID-19 Ag Rapid Test Device, was commercialized by Abbott Rapid Diagnostics Jena GmbH at the beginning of October 2020. Successively, the STANDARD Q COVID-19 Ag Test was developed by SD Biosensor and was World Health Organization Emergency Use Listing (WHO EUL) certified [115]. Another commercially available antigen test, called Sofia SARS Antigen Fluorescent Immunoassay, was developed by Quidel. It is based on advanced immunofluorescence-based lateral flow technology in a sandwich design for qualitative detection of N-protein from SARS-CoV-2 [116]. The Sofia SARS Antigen FIA provides automated results in 15 min and was issued Emergency Use Authorization (EUA) by the FDA.

These tests allow qualitative detection of the virus in the human nasopharynx within 15–30 min with excellent specificity (100%), but their sensitivity is lower compared with RT-PCR methods. Therefore, several studies are in progress to increase the accuracy, the sensitivity and the detection throughput of the aforementioned tests.

The main analytical characteristics of the described PBBs for SARS-CoV-2 antigen detection are summarized in Table 2.

### 3.3. SARS-CoV-2 Antibody Detection

Several LFIAs have been realized for the detection of serum antibodies in patients who have been exposed to the SARS-CoV-2 virus (serological tests). It has been recently demonstrated that seroconversion in patients generally starts after a week of the first symptom [117]. This time gap may lead to false negative responses: the patient can be infected but has not yet produced the antibodies at a detectable level. It has been found that IgM levels increase after 2–3 days of the first symptom, reach their peak after 2 weeks and then rapidly decrease within 3 weeks. IgG levels increase 10–14 days after symptom onset but remain at a higher concentration even after 7 weeks [117]. Therefore, serological testing must be carried out at least 14 days after symptom onset to avoid false negative results.

Another big issue with serological tests is their cross-reactivity because of the similarities of the target antigen to different antigens, which can lead to false positives. The specificity of the antigen used to capture the antibodies is a very important issue for the efficacy of LFIA tests. It has been found that the specificity and of the S1 subunit is higher compared with the nucleocapsid (N) protein and the native state S-trimer protein for SARS-CoV-2 antibody capture [27,118]. Moreover, the S1 subunit showed higher sensitivity than the N-protein and the receptor-binding domain (RBD), which would make the S1 subunit the best candidate for large-scale serological tests and for the evaluation of SARS-CoV-2 vaccines that are currently in development.

Despite these limitations, serological immunoassays are a fast and cheap method for rapid screening of previous SARS-CoV-2 infection, and represent an important tool for vaccine and epidemiological studies.

Several LFIA kits are actually available on the market for the detection of IgG and/or IgM antibodies for SARS-CoV-2, but some of them are waiting for legal approval. 

Li et al. developed quick paper-based lateral flow tests strips (LFIAs) for the combined detection of IgM and IgG antibodies with a visual readout [119]. The assay consists of immobilizing SARS-CoV-2 N-protein to the strip’s surface and coupling anti-human IgG with gold nanoparticles (AuNPs). When IgG antibodies are present in the sample, the antibody binds to the anti-human IgG monoclonal antibodies present in the conjugation pad and the complex migrates to the SARS-CoV-2 N-protein bound in the test line, showing a visible color. At the same time, the unbound conjugates bind to the goat anti-mouse IgG polyclonal antibody on the control line. The POC assay takes place in 15 min and shows good specific and stability, comparing the results with PCR. A similar LFIA test strip was successively developed by Wen and coworkers for IgG detection only [120].

In another study, lanthanide doped polystyrene nanoparticles (LNPs) were integrated into a SARS-CoV-2 LFIA to detect IgG in human serum [121]. A recombinant N-phosphoprotein was added to a nitrocellulose membrane as test line for specific IgG capture. LNPs were used as a fluorescent reporter labeling the mouse anti-human IgG antibody to capture the targets. Goat anti-rabbit IgG was used as the control line. The fluorescence signal at 615 nm was measured by a portable fluorescence reader and produced a bright fluorescent zone on the strip. Moreover, the developed assay showed good sensitivity, reproducibility and diagnostic accuracy, and, therefore, it can be useful for monitoring the progression of COVID-19 and for evaluation of a patient’s response to a specific treatment.

LFIAs can be used also to detect SARS-CoV-2 “total antibodies”, as described by Cavalera and co-authors, who developed a POC multi-target LFIA enabling the specific and sensitive detection of total immunoglobulins (IgG, IgM, IgA) directed towards the N-protein of SARS-CoV-2 [122]. Specific anti-SARS-CoV-2 antibodies are revealed by a colorimetric probe formed by N-protein and gold NPs through a visual readout. The assay showed 95% sensitivity and 100% diagnostic specificity, by agreeing with a reference ELISA method.

It is well known that the SARS-CoV-2 specific response includes IgG, IgG and IgA [123]. In a recent study, it seemed that IgA can be detected earlier than IgM and IgG antibodies, reaching its peak in the third week, and maintains a stronger and more persistent response than IgM and IgG. Moreover, IgA’s response seems to be more potent than IgG’s response in neutralizing SARS-CoV-2. For these reasons, IgA might have a potential role during early SARS-CoV-2 infection and, therefore, its detection could be crucial for earlier diagnosis [123].

To this end, Roda et al. developed a LFIA for selective detection of IgA in the serum and saliva of COVID-19 patients [124]. A recombinant N-antigen is specific for the capture of SARS-CoV-2 antibodies, and a labeled anti-human IgA reveals the bound IgA fraction. The immunosensors are based on both optical and chemiluminescence (CL) detection, using nanoAu-labeled anti-human IgA and measuring the light signal resulting from the reaction between horse radish peroxidase (HRP) - labeled anti-human IgA and H_2_O_2_/luminol/enhancer substrate, respectively. In particular, the colorimetric immunosensor was coupled with a smartphone reader and the CL immunosensor with a portable CCD (charge-coupled device) camera, which provided higher sensitivity. To date, this is the only LFIA for detecting salivary IgA. This assay could be extremely useful for massive noninvasive monitoring of the early SARS-CoV-2 immune response.

Up to now, hundreds of LFIA kits for SARS-CoV-2 antibody detection in serum or plasma have entered the market, mostly commercialized by companies from China and the US. The Assure COVID-19 IgG/IgM Rapid Test Device was the first US FDA-authorized test for emergency use in July 2020. At present, not all of them have received Emergency Use Authorization (EUA) by the US FDA or by other drug regulatory authorities. They show an average of about 80% sensitivity for IgM and 97% for IgG.

Table 3 summarizes the main analytical characteristics of the described PBBs for SARS-CoV-2 antibody detection.

### 3.4. μPADs for COVID-19 Detection

μPAD methods are being developed to provide a rapid and cost-effective solution to POC needs during the COVID-19 pandemic. They are realized by integration of the microfluidics technique with paper-based detection approaches to obtain improved fluidic control and enhanced test performance [125].

Up to now, there have been only two paper-based microfluidic devices for SARS-CoV-2 detection reported in the literature.

The first one is a microfluidic platform for the semi-automatic selective detection of IgG and IgM from up to 50 different serum samples in parallel [126]. The device shows several advantages, such as minimal assay reagent consumption, multiple analyte detection, high sensitivity (95%), high specificity (91%) and high-throughput, but it suffers from drawbacks such as cross-reactivity and the long total analysis time. For 50 assays, the total assay running time is 5.5 h (6.6 min per assay). However, the overall good performance achieved by this device makes its use very interesting for low-cost massive screenings.

In the second work, Ramachandran and co-workers combined an electrokinetic microfluidic technique called isotachophoresis (ITP) for automated purification of target RNA with RT-LAMP and the CRISPR assay for the detection of SARS-CoV-2 RNA from clinical nasopharyngeal swab samples [127]. The proposed method takes about 35 min to give a fluorescence result from a row sample, a significant improvement compared with existing CRISPR-based methods for COVID-19. The authors reported that the proposed device is the fastest CRISPR-based method for the detection of SARS-CoV-2 with clinically relevant specificity and sensitivity. However, in this study, the RT-LAMP step was carried out outside the chip and, therefore, future work must be done to integrate all assay steps in a portable microfluidic system.

The main analytical characteristics of the two reported μPADs for SARS-CoV-2 antibodies and gene detection are summarized at the end of Table 3 and Table 1, respectively.

## 4. Conclusions and Future Perspectives

This review deals with the recently developed PBBs for COVID-19 detection. The simplicity, rapid response, low cost and portability of these biosensors play a crucial role for their POC application, providing great advantages for use by nonclinical individuals in their homes, especially in developing countries.

There has been a continuous improvement in LFA techniques, leading to increased sensitivity and specificity. New approaches are in development, such as signal amplification by the introduction of nanomaterials with novel physicochemical properties [45,67], or specific enzymes [128] in the assay platform, sample concentration [60,129] and fluidic control strategies [130,131,132].

The recent use of isothermal amplification in CRISPR, NALF and NALFIA methods for SARS-CoV-2 gene detection have helped to overcome the problem of false negative results. In particular, the CRISPR method, coupled with LFA, shows great promise in achieving comparable sensitivity and specificity to PCR methods, together with a faster assay time (about 1 h), unlike PCR tests, which typically take 4–6 h to complete [102,103]. For these reasons, CRISPR-LFA represents a cheap and promising alternative method for SARS-CoV-2 gene detection.

Another aspect that strengthens the value of PBBs is their capability for multiplexing, allowing the detection of several COVID-19 biomarkers simultaneously [48]. In particular 3D μPADs are particularly promising, thanks to the network of microchannels that enables multiple functions [91,92]. Multiplex detection significantly improves assay productivity and accuracy, and the reliability of the diagnostic tests, providing better monitoring of the evolution of the disease, often involving a panel of biotargets. Furthermore, taking into account the possibility that the COVID-19 infection will remain endemic in the population, like common flu viruses, a multiplex testing method for multiple diseases could become a backbone for a routine testing platform in future.

A critical issue of LFA-based serological tests is related to the cross-reactivity of SARS-CoV-2 with seasonal coronaviruses, which can generate false positive results. In particular, it has been found that the cross-immune reactivity mainly targets the S-trimer protein and the 1AB polyprotein, which are the regions with high sequence similarities between low-pathogenic human coronaviruses and SARS-CoV-2. Therefore, by using the S1 subunit, which does not cross-react with circulating human coronaviruses, instead of the S-trimer, it is possible to avoid cross-reactivity, thus increasing the specificity of the serological tests. Moreover, further studies are in development to establish whether cross-neutralizing activity also exists, with the consequent cross-protective effects [133].

The biggest disadvantage of PBBs is that all tests are mainly qualitative or semi-quantitative. This issue can be overcome by integrating the PBBs with emerging smartphone technology. Nowadays, smartphones are multitasking devices which represent a perfect platform for POC and point-of-need biosensor development. They can be used as a camera allowing signal readout. The majority of embedded systems use smartphone cameras as “smart detectors” for colorimetry, fluorescence, absorbance and chemiluminescence. The images are successively analyzed using a dedicated image-processing smartphone app, allowing analyte detection in a few minutes. The smartphone app can be implemented using the Internet of Medical Things (IoMT). The results can be easily shared with a doctor, who provides the patient with the proper treatment at while he/she is at home, and with healthcare authorities, allowing easier monitoring of the spread of the disease, as well as epidemiological surveillance [70,134,135].

The PBBs for SARS-CoV-2 gene, antigen and antibody detection described in this review gave some false negative and positive results. In particular, PBBs for gene detection generally give false negatives, caused by possible mutations which can occur in the SARS-CoV-2 genome, or by the lack of amplification methods. These drawbacks can be overcome by simultaneous detection of multiple regions of the SARS-CoV-2 genome [136] and by introducing the isothermal amplification method in CRISPR, NALF and NALFIA systems. Further studies will be necessary to overcome the false negative results obtained with the PBBs for antigen detection, which can be ascribed to the low viral load in the nasopharyngeal swab samples. In these cases, negative results from the antigen test may need to be confirmed with a molecular PCR test prior to making treatment decisions or to prevent the possible spread of the virus due to a false negative. As for the PBBs for antibody detection, they give both false negative and false positive results, the first being related to the seroconversion time and the second to the antibodies’ cross-reactivity phenomena. It is of extreme importance to wait the proper time gap after seroconversion before testing and to use the S1 subunit with low sequence similarity to other coronaviruses as the antigen target region, as reported above. Table 4 summarizes the potential causes of false positive and false negative results, and the recommended measures to minimize them.

In less than 1 year since the start of the pandemic, a large number of LFA-based serological tests for COVID-19 antibody detection have been granted an EUA by the FDA and have already entered the market, whereas a lower number of antigen tests have been commercialized. However, the quality of both tests needs to be re-evaluated, particularly the antigen test, because of the occurrence of many false negative results due to the low viral load present in clinical samples of asymptomatic patients or patients after weeks of infection. New studies will be focused on increasing the sensitivity and detection throughput of such tests for their commercial success as rapid clinical diagnostics for massive screening and monitoring.

Nevertheless, it is envisioned that further efforts in the development of PBBs with improved performance, combined with smartphones and IoMT applications, will provide new insights into designing more versatile, user-friendly and robust COVID-19 diagnostics for mass testing, thus improving the healthcare system to manage the actual pandemic and similar diseases.

## Figures and Tables

**Table 1:** Severe acute respiratory syndrome coronavirus 2 (SARS-CoV-2) gene detection methods. Abbreviations: RT-PCR = reverse transcription polymerase chain reaction; RT-LAMP = reverse transcription loop-mediated isothermal amplification; RPA = transcription-recombinase aided amplification; RT-RAA = recombinase polymerase amplification.

PBB Type	Biomarker	Amplification Method	Readout	LOD	Sensitivity and Specificity	Assay Time	POC	Ref.
NALF	RdRp, ORF3a, N gene	RT-PCR	fluorescence	10 copies/test for each gene	100% 99%	30 min	no	98
NALFIA	ORF1ab, N gene	RT-LAMP	colorimetric	12 copies/reaction	100% 100%	1 h	yes	99
NALFIA	ORF1ab, E gene N gene	none	fluorescence	500 copies/mL	100% 99%	<1 h	yes	101
CRISPR-Cas12	E gene N gene	RT-LAMP	fluorescence,visual	10 copies/μL	95% 100%	~45 min	no	106
CRISPR-Cas12	ORF1a, ORD1b, N gene, E gene	RT-RAA	fluorescence	-	100%	<1 h	yes	107
CRISPR-Cas12	RdRp, ORF1b,ORF1ab	RPA	fluorescence,visual	10 copies/μL	-	90 min	yes	108
CRISPR-Cas13	S gene ORF1ab	RPA	fluorescence	10–100 copies/μL	-	<1 h	yes	109
CRISPR-Cas13	N gene ORF1ab	RT-LAMP	fluorescence,	6.75 copies/μL 6\1	-	1 h	yes (commercially available)	110
μPAD	RNA	RT-LAMP	fluorescence	-	-	30 min	no	127

**Table 2:** SARS-CoV-2 antigen detection methods.

PBB Type	Biomarker	Amplification Method	Readout	LOD	Sensitivity and Specificity	Assay Time	POC	Ref.
LFIA	N-protein antigen	no	Fluorescence	-	68% 100%	10 min	yes	113
LFIA	N-protein antigen	no	fluorescence, visual	0.65ng/ml	-	-	no	114

**Table 3:** SARS-CoV-2 antibody detection methods.

PBB Type	Biomarker	Amplification Method	Readout	LOD	Sensitivity and Specificity	Assay Time	POC	Ref.
LFIA	IgM/IgG	none	visual	-	-	15 min	yes	119
LFIA	IgM/IgG	none	visual	-	-	15 min	yes	120
LFIA	IgG	none	fluorescence	-	-	-	yes	121
LFIA	IgM/ IgG/IgA	none	fluorescence	-	94.6% 100%	-	yes	122
LFIA	IgA (also in saliva)	none	colorimetric/chemiluminescence	-	-	15 min	yes	124
μPAD	IgG/IgM	none	fluorescence	-	95% 91%	6.6 min/assay 5.5 h/50 assays	yes	126

**Table 4:** Potential causes of false positive and false negative results with PBBs for COVID-19 detection, and the recommended measures to minimize them.

Target Analyte	Type of Result	Cause	Solution
gene	false negative	possible mutations in the SARS-CoV-2 genome	simultaneous detection of multiple regions of the SARS-CoV-2 genome
	false positive false negative	cross-reactivity of SARS-CoV-2 with other coronaviruses lack of amplification	simultaneous detection of multiple regions of the SARS-CoV-2 genome use of isothermal amplification in CRISPR, NALF and NALFIA methods
antigen	false negative	low viral load in the nasopharyngeal swabs	-
antibodies	false negative false positive	seroconversion cross-reactivity of SARS-CoV-2 with other coronaviruses	waiting the right time gap before testing using subunit S1 instead of the S-trimer protein which has high sequence similarities with other coronaviruses
